# Leveraging best practices: protecting sub-Saharan African prison detainees amid COVID-19

**DOI:** 10.11604/pamj.2020.36.121.24133

**Published:** 2020-06-24

**Authors:** Batholomew Chireh, Samuel Kwaku Essien

**Affiliations:** 1 Saskatchewan Health Quality Council, Saskatoon, Saskatchewan, Canada; 2 School of Rehabilitation Science, University of Saskatchewan, Saskatoon, Saskatchewan, Canada

**Keywords:** COVID-19, novel coronavirus, 2019-nCoV, sub-Saharan African countries, prisoners

## Abstract

The risk of infection and death from COVID-19 is higher among older prisoners with pre-existing health conditions especially in sub-Saharan African. Hawks L *et al*. raise four concerns that need to be considered when developing public health and clinical responses to COVID-19 to protect prisoners. This paper applies these concerns to the sub-Saharan African context. These focus areas include 1) challenges of social distancing; 2) higher risk of severe infection and death; 3) difficulties health care systems may face in the case of COVID-19 surge; and 4) recommended solutions to prevent harm and preventing a public health catastrophe. Prisoners are more vulnerable and the time to take immediate actions to minimize an imminent COVID-19 outbreak and its impacts is now.

## Commentary

Most prisons in sub-Saharan African countries are overcrowded with prisoners living in extremely dirty and unpleasant conditions. In 2016, an estimated 668,000 people were incarcerated and actively serving prison sentences [1]. Another report estimated that 50% to 90% of detainees constitute pretrial detainees of the total prison population of most countries in the continent. Which means, most prisons in Africa can be decongested if there is the political will [2]. Amid the coronavirus pandemic, prisoners are among the vulnerable groups who are at a higher risk of contracting the deadly disease. Systemic poor prison conditions will worsen the plight of most prisoners in this COVID-19 pandemic era. Extreme poor healthcare systems inside prisons do not provide the necessary medications and health personnel for the treatment of prisoner´s basic health needs making them vulnerable to COVID-19 risk. This risk is further exacerbated among older prisoners with underlying medical conditions such as cardiovascular disease, diabetes, chronic respiratory disease, and cancer [3]. Evidence also shows that institutions such as mines and prisons in which people live in proximity can act as incubators of infection [3]. There are already hundreds of COVID-19 cases in Sub-Saharan Africa prisons. For instance, in Cameroon, Guinea, or South Africa, the detention centers are quickly becoming epicenters of the pandemic [2]. COVID-19 calls for states to quickly solve issues regarding their detention system to avoid turning detention centers to epicenters of the outbreak. Countries like Burkina Faso, Cameroon, Cote d´Ivoire, Democratic Republic of Congo (DRC), Ghana, Mozambique, Niger, Nigeria, Senegal, Tanzania, and Togo have already taken several measures to free up space in prisons [2]. However, most of the measures are still insufficient to prevent an outbreak. A recent publication by Hawks L *et al*. [4] suggests that prison conditions have important implications for how public health and clinical responses should be developed towards the COVID-19 fight. Therefore, sub-Saharan African countries must take note of the concerns discussed below and take precautionary measures to prevent the spread of COVID-19 among this vulnerable population. Hawks L *et al*. [4] discuss four areas of concern to consider when developing public health and clinical responses to protect prisoners from COVID-19. Although prison conditions in the United States may be quite different from those of sub-Saharan African countries, this paper applies these focus areas to the African context and provides simple and immediate measures to proactively prevent the spread of COVID-19 among prisoners in the region.

The first area of concern that relates to countries in Sub-Saharan Africa as well as other low and middle countries is the challenge of social or physical distancing. The COVID-19 outbreak on the Diamond Princess Cruise ship highlights the importance of social distancing in the fight against COVID-19. For example, within 4-weeks, 700 people got infected and 12 casualties were recorded out of the total number of 3700 passengers and crew held onboard the ship. The major source of infection was through kitchen staff housed together and was responsible for feeding passengers on board [5]. Similar conditions are common and conducive for COVID-19 spread in most African prisons given the poor infrastructure of prisons in the region. Also, the fact that prison officers, other staff, and relatives of incarcerated persons often visit prison facilities and then return, it exposes inmates to a higher risk of COVID-19. Social distancing may be a practical impossibility considering the crowded nature of most prisons in the region. The second area of concern is that prisoners are at high risk of severe infection and death. As stated earlier older prisoners and persons with underlying medical conditions such as cardiovascular disease, diabetes, chronic respiratory disease, and cancer are more likely to report severe infection and death in low- and middle-income countries [3]. While statistics on the average age and duration of pretrial detention in Africa are difficult to obtain, evidence suggests that waits are longest in Central and West African nations. For example, nearly the highest rate of pretrial prisoners in prison in the world is found in Liberia (97.3 percent), the second highest in the world is Mali with 88.7 percent, Benin is 4^th^ with 79.6 percent, and Niger 5^th^ with 76 percent [1]. This leads to overcrowding and possible disease spread in SSA prisons. Health data from African prisons differed by regional blocks. A study published in The Lancet found that comorbid HIV and tuberculosis prevalence among prisoners in Africa ranges between 2.3% to 34.9% with a regional breakdown of 2.3% to 10.8% in West Africa and 4.2% to 23% in East Africa [1]. Also, a recent Cameroonian study reported higher levels of diabetes, hypertension, and obesity among prison inmates [6]. All of which are risk factors for an increased risk of COVID-19 deaths.

Thirdly, the effects on health care systems in an imminent COVID-19 outbreak in SSA prisons are of concern. Low-and middle-income countries (LMICs), with fragile healthcare systems, are already stretched by the dual burden of non-communicable diseases (NCDs-such as type 2 diabetes, cancers, arthritis, hypertension, stroke, heart diseases,) and earlier infectious diseases [1,6]. Not only are these diseases increasing, but they are increasing disproportionately among women, rural residents, prisoners, and those with low income [1,6]. These fragile health care systems may not be able to contain a surge in COVID-19 in the general population as well as the prison population. The current COVID-19 pandemic if not well managed in both the general population and in prisons has the potential to cause similar havoc just like the recent Ebola virus disease outbreak in some West African countries. The inability of less-resourced African prison systems to bear hospital-related costs may be a disincentive for referring inmates with pre-existing conditions to seek better health care although it is their right. The fourth area of focus is to design strategies to prevent a public health catastrophe in SSA prisons. Even before the advent of COVID-19, civil society and other civil rights organizations have strongly advocated for the provision of basic health amenities such as personal protective equipment, routine screening or testing, and availability of medical care for inmates [2].

In this commentary, we sought to leverage best practices from other jurisdictions that have proven to have yielded positive results in the fight against infectious diseases in prisons and apply them to the African context. We therefore recommend the following solutions. First and foremost is the decongestion of prison populations by releasing non-violent inmates 50+ years with serious health issues and reducing unnecessary pretrial detention. While the COVID-19 may be an albatross around the necks of most SSA countries, it opens a window of opportunity for the respective countries to decongest their prisons and drastically reduce prison populations to avoid a looming outbreak. This has been found as the most effective way of combating COVID-19 in prisons [7]. Lessons can be drawn from the US and other developed countries where prison programs such as probation, conditional sentences, provincial parole, and community alternative to remand or reduction in pretrial detention have shown to have effectively decongested prisons in these countries [8]. Secondly, for those remaining in the detention facilities, they should be allowed access to mental health professionals and provided with a standard of healthcare that meets each prisoner´s individual needs similar to those available in the community, and that ensures the maximum possible protection against the spread of COVID-19. A recent systematic review in the US found that improving health in people in jails and prisons can also improve the health of the general population, improve the safety of communities, and decrease health care costs. These researchers believed that Emergency Department use will be reduced if infectious diseases can be treated to limit ongoing transmission, while crime rates can be decreased through the treatment of people with mental illness, and provision of quality and accessible primary health care [9].

Thirdly, ensure that prisoners have separate cells, giving them the ability to physically distant from each other. Most prisons in SSA do not have the minimum space requirements for their inmates mainly due to high rates of pretrial detentions and limited infrastructure [1]. This can only happen if prisons are decongested. Although this may be practically difficult to achieve in most prisons in SSA countries at this time, a recent systematic review of studies from 16 countries found that COVID-19 spread was lower with the physical distancing of 1 meter or more, compared with a distance of less than 1 meter in all populations [10]. Finally, we recommend the heightening of sanitation standards for all staff, inmates, and facilities, ensuring ample sinks for proper handwashing. As handwashing and sanitation are key to preventing the virus´ spread, free supplies are public healths imperative. Good sanitary conditions especially handwashing remains one of the most effective ways of preventing COVID-19 spread. Before the emergence of Coronavirus (COVID-19), proper handwashing was regarded as the most effective means of preventing the spread of other viral illnesses such as flu and cold. The spread of earlier outbreaks such as SARS (severe acute respiratory syndrome) and MERS (Middle East respiratory syndrome) were also reduced through frequent and proper handwashing. That is why it is also recommended for the prevention of Coronavirus (COVID-19). This should be highly encouraged in detention centers in SSA countries

In conclusion, this paper discusses four areas of concern by Hawks L *et al*. [4] that needs to be considered when developing public health and clinical responses to COVID-19 to protect prison inmates in general, in the context of SSA countries and provides simple and immediate measures to proactively prevent the spread of COVID-19 among prisoners. These focus areas include but do not limit to 1) challenges of social or physical distancing in SSA prisons; 2) prisoners at higher risk of severe infection and death; 3) considering the difficulties health care systems may face in the case of a COVID-19 surge in these countries, and; 4) recommended solutions to prevent harm and preventing a public health catastrophe through; decongestion of prison populations by releasing non-violent inmates 50+ years with serious health issues and reducing unnecessary pretrial detention, access to mental health professionals and provision of standard healthcare, ensuring prisoners have separate cells, giving them the ability to physically distant from each other and heightening of sanitation standards for all staff, inmates, and facilities, ensuring ample sinks for proper handwashing.
